# MxiA, MxiC and IpaD Regulate Substrate Selection and Secretion Mode in the T3SS of *Shigella flexneri*

**DOI:** 10.1371/journal.pone.0155141

**Published:** 2016-05-12

**Authors:** Da-Kang Shen, Ariel J. Blocker

**Affiliations:** 1 School of Cellular & Molecular Medicine, Faculty of Biomedical Sciences, University of Bristol, Bristol, United Kingdom; 2 Schools of Cellular & Molecular Medicine and Biochemistry, Faculty of Biomedical Sciences, University of Bristol, Bristol, United Kingdom; Centre National de la Recherche Scientifique, Aix-Marseille Université, FRANCE

## Abstract

Type III secretion systems (T3SSs) are central virulence devices for many Gram-negative bacterial pathogens of humans, animals & plants. Upon physical contact with eukaryotic host cells, they translocate virulence-mediating proteins, known as effectors, into them during infection. T3SSs are gated from the outside by host-cell contact and from the inside via two cytoplasmic negative regulators, MxiC and IpaD in *Shigella flexneri*, which together control the effector secretion hierarchy. Their absence leads to premature and increased secretion of effectors. Here, we investigated where and how these regulators act. We demonstrate that the T3SS inner membrane export apparatus protein MxiA plays a role in substrate selection. Indeed, using a genetic screen, we identified two amino acids located on the surface of MxiA’s cytoplasmic region (MxiA_C_) which, when mutated, upregulate late effector expression and, in the case of MxiA_I674V_, also secretion. The cytoplasmic region of MxiA, but not MxiA_N373D_ and MxiA_I674V_, interacts directly with the C-terminus of MxiC in a two-hybrid assay. Efficient T3S requires a cytoplasmic ATPase and the proton motive force (PMF), which is composed of the ΔΨ and the ΔpH. MxiA family proteins and their regulators are implicated in utilization of the PMF for protein export. However, our MxiA point mutants show similar PMF utilisation to wild-type, requiring primarily the ΔΨ. On the other hand, lack of MxiC or IpaD, renders the faster T3S seen increasingly dependent on the ΔpH. Therefore, MxiA, MxiC and IpaD act together to regulate substrate selection and secretion mode in the T3SS of *Shigella flexneri*.

## Introduction

Type III secretion systems (T3SSs) are central virulence devices for many Gram-negative bacterial pathogens of humans, animals & plants. They translocate virulence proteins into eukaryotic host cells to manipulate them during infection. T3SSs are key to the virulence of enteric pathogens such as *E*. *coli*, *Salmonella* and *Shigella* species. Bacterial diarrheal illnesses lead to ~10% of deaths in children <5 yrs in the developing world [1]. *Enterobacteriaceae* also lead to serious disease outbreaks in rich societies.

*Shigella* is the agent of human bacillary dysentery. Its T3SS consists of a cytoplasmic portion and a transmembrane region traversing both bacterial membranes, into which a hollow needle is embedded protruding from the bacterial surface [2]. Physical contact with eukaryotic host cells activates the secretion system, which initiates secretion and leads to formation of a pore, formed by the bacterial proteins IpaB & IpaC in *Shigella*, in host-cell membranes [3]. Through the needle [4] and pore channels, the effectors are translocated to facilitate host cell invasion [3]. The needle tip complex (TC), which contains IpaD and IpaB, is the host cell sensor and transforms itself into the translocation pore [5, 6] via addition of IpaC upon secretion activation [7, 8]. IpaD is hydrophilic and required for tip recruitment of the other two proteins, which are hydrophobic. Together, they are called the translocators.

Effectors acting late in the host cell manipulation cascade are only expressed once presynthesised early effectors have been secreted at host cell contact, but via differing mechanisms from one T3SS-carrying organism to another [9]. Yet, one regulatory cascade is conserved, that allowing hierarchical secretion of substrates [10]. How this cascade functions in animal pathogens is the focus of this work. There, effector secretion is initially prevented through concerted action of surface TC proteins and regulators controlling secretion from within the bacterial cytoplasm. The TC may prevent premature effector secretion by allosterically constraining the T3SS in a secretion “off” conformation [11, 12]. Upon physical contact of the TC with host cells [13], a signal is transmitted via the TC [13] and needle [14] to the cytoplasm where it triggers secretion [11]. Next, translocators are secreted to form the pore in the host membrane. Successful pore formation at the needle tip generates a second needle-transmitted signal [11, 14, 15], allowing inactivation or T3S-mediated removal of a conserved cytoplasmic regulatory protein, MxiC in *Shigella* [16]. Third, early effector proteins are secreted and translocated into the host cell [11] and expression of late effectors, including the family of *ipaH* genes [17], is activated via activation of the transcription factor MxiE [18].

Members of the MxiC family [19] are termed “gate-keepers” as they regulate initial substrate selection hierarchies. In animal pathogens, all MxiC-class proteins prevent effector secretion in the absence of an activation signal: when *mxiC* or its homologues are deleted, bacteria constitutively secrete effectors [16]. Deletion of *mxiC* and some of its homologs also leads to decreased translocator secretion [11], indicating the proteins can also promote translocator secretion. The gate-keepers have conserved structures [20, 21]: after an N-terminal secretion signal and chaperone binding domain (CBD), three α-helical X-bundles form a flat and elongated structure [21] typical for “hub proteins” regulating processes via interaction with multiple partners. In some species, gate-keepers are composed of two proteins where the second polypeptide covers the C-terminal X-bundle (domain 3; [20]).

Several gate-keepers bind heterodimeric intrabacterial chaperones [20, 22] but, no chaperone was found in *Shigella* [16, 23]. Some MxiC homologues also interact with effector proteins and this can help regulate the secretion hierarchy [24]. However, none of these partners are conserved, making them unlikely mediators of conserved gate-keepers functions. Instead, MxiC homologs may control secretion by interacting with the cytoplasmic face of the T3SS export apparatus (EA), blocking an acceptor site for effectors and/or altering its activity [11, 12, 15, 25, 26].

**Fig 1:**
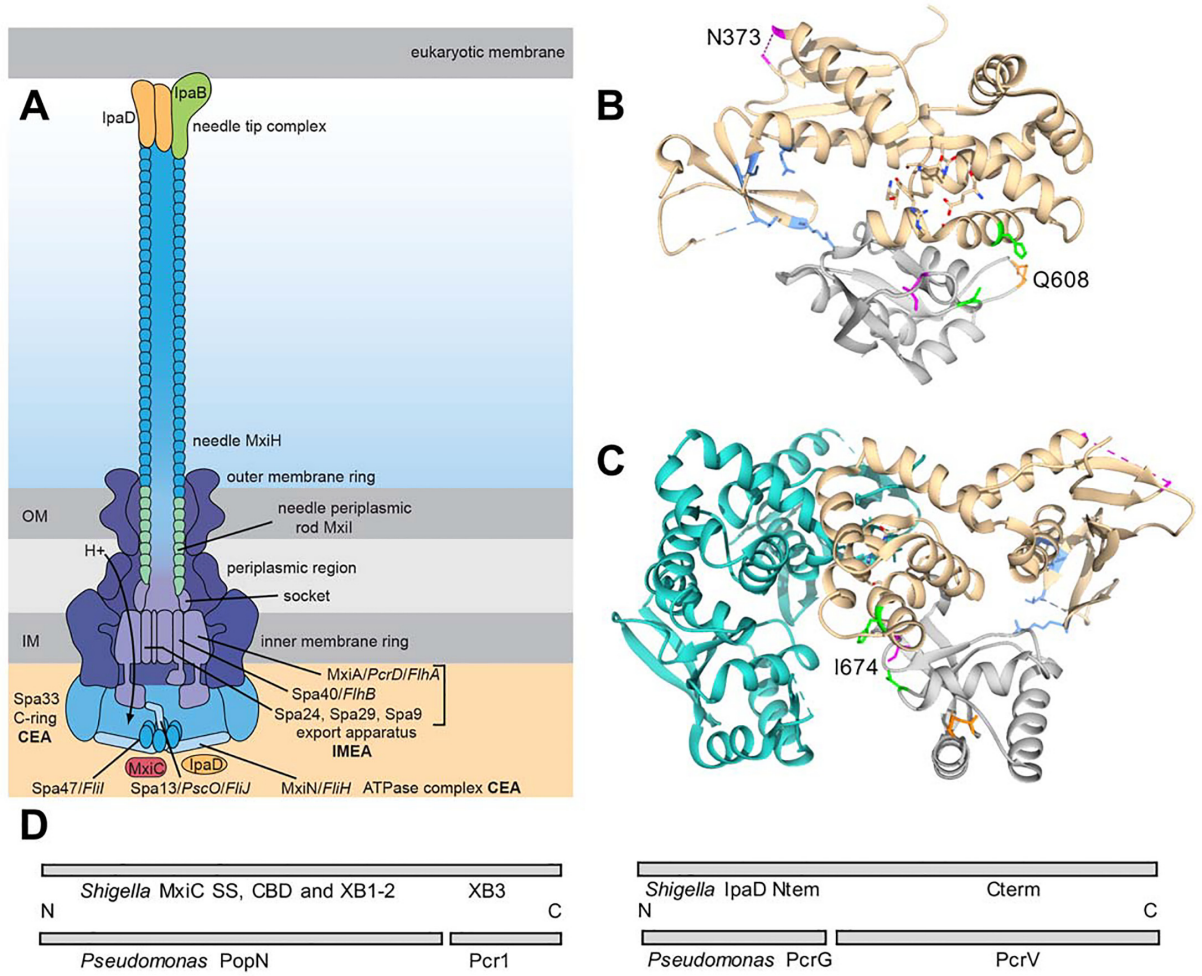
Schematic of *Shigella* T3SS and location of MxiA_C_ mutations altering substrate selection. (A) Shematic of *Shigella* T3SS. Needle components in blue-greens, transmembrane base dark purple, IMEA light purple, CEA components (C-ring and ATPase complex) in light blues. A curved arrow shows the proposed direction of proton flow during protein export, which may be coupled to ATP hydrolysis. *Pseudomonas* vT3SS and *Salmonella* fT3SS homologs mentioned in the text are shown in italics. (B) MxiA_C_ monomer (chain B [36]), putative oligomerisation domain (indicated by aas with charges shown) facing viewer. *Grey*, domain 4, facing cytoplasm; *light blue*, aa involved in chaperone binding within domain 2; mutations in both of these affect substrate selection in FlhA [44, 45]. *Pink*, location of mutations found in this work; *top right* N373, in an unstructured loop; *bottom*, I674. *Orange*, Q608 and *green*, location of two activating mutations identified by Rietsch [31] (homologous to Y587 and M667 in PcrD). Mutations in pink and orange aas are all surface located and affect interaction with MxiC. *Right*: *cyan*, chain A of oligomer. Assembly rotated left to show mutations do not cluster at oligomer interfaces (inside of nonamer ring facing viewer). (D) Schematic presenting the sequence homologies and functional correspondences between *Shigella* and *Pseudomonas* regulatory proteins mentioned in this work. Proteins are presented linearly, from N- to C-terminus; SS, secretion signal; CBD, chaperone binding domain; XB, X-bundle.

The conserved EA is composed of 5–6 inner membrane proteins (IMEA) and some peripheral/cytoplasmic ones (CEA) (Fig 1A). The latter includes 1–2 “C-ring” components that bind cytoplasmic IMEA portions and 3 components of an ATPase complex interacting with the IMEA and C-ring and evolutionarily related to F1Fo-ATPases [27]. F1-ATP synthase’s hexameric ring connects to Fo via a central stalk, rotated for catalysis through the ring by proton motive force (PMF)-driven rotation of Fo in the membrane. A peripheral stalk connecting F1 and Fo functions as a stator. The flagellum, which also uses a T3SS (fT3SS) to assemble, and the *Pseudomonas* and *Yersinia* virulence mediating T3SS (vT3SS), all use the PMF to catalyse protein export [28–31]. In the fT3SS, IMEA part(s), including FlhA, function as a proton-protein antiporter [32], the activity of which is initiated and/or stimulated by the hexameric ATPase FliI [33, 34]. FliI’s activity is coupled to the IMEA’s by FliJ’s interaction with FlhA [32]. FlhA, known as MxiA in the *Shigella* vT3SS, is a polytopic membrane protein with a large C-terminal transmembrane region essential for the secretion process [35], which may form a nonameric ring within the IMEA [36]. It binds FliJ, Spa13 in the *Shigella* vT3SS, via an extended linker region connecting the cytoplasmic region to the membrane [32, 37]. FliJ shows structural similarity to the rotating subunit of F1Fo-ATPases [38], whilst FliH, probably MxiN in the *Shigella* vT3SS, is homologous to their stator region.

The only IMEA components so far involved in substrate selection are those of the FlhB and FlhA families. In FlhB homologs, an autocleavage/conformational change allows export of the next appropriate substrates upon base completion [27, 39]. FlhA family proteins suppress premature export of these substrates [27, 31] and may then selectively stimulate their export [40, 41] (Fig 1B and 1C). Yet, no MxiC homolog was identified in fT3SSs. But, in several vT3SSs, a MxiC homologue fraction is membrane-associated [24, 42]. In one case, its departure from this location correlates with effector secretion [26]. MxiC also interacts with the ATPase and the needle periplasmic rod [15, 16], although its action(s) on them is undefined. In addition, a “sorting platform” composed of C-ring and ATPase components is selectively “loaded” with translocators and effectors through action of their chaperones and a MxiC homolog [43]. Recently, *Pseudomonas* Pcr1, a homolog of MxiC’s domain 3 (Fig 1D), was found to interact directly with PcrD, an FlhA homolog [31].

MxiC homologues also interact with the hydrophobic translocators’ chaperone, IpgC in *Shigella* [15, 42], via a domain 3 site [22, 46]. Furthermore, alteration of IpaD’s self-chaperone domain [13] or, in other species, its separately encoded intrabacterial chaperone [12], upregulates translocator export. As chaperones are not secreted, the latter control gate-keeper activity intrabacterially [12]. This is supported by genetic dissociation of IpaD’s intracellular and TC regulatory roles [13]. IpaD’s intracellular role also requires MxiC [11, 13]. This suggests all these factors interact intrabacterially to prevent premature translocator secretion. Indeed, PcrG, the *Pseudomonas* equivalent of the IpaD self-chaperone domain (Fig 1D), interacts with Pcr1 [31]. Furthermore, MxiC, IpaD, IpgC, IpaB and IpaC interact, albeit weakly [23].

IpaD removal from the cell increases the secretion rate. *ΔipaD* is one of only two mutants identified as a “fast” constitutive secreter in *Shigella*, the other being *ΔipaB* [5]. By comparison, the constitutive secretion observed in *ΔmxiC* is slower [11]. Lack of IpaB liberates IpgC, a transcriptional co-activator of late effectors genes in *Shigella*. However, IpaB is present at wild-type levels in *ΔipaD* [23], indicating the cause of rapid secretion in *ΔipaD* lies elsewhere. It may result from absence of an inhibitory complex containing IpaD associated with the secretion apparatus. Indeed, *Pseudomonas* PcrG interacts with PscO, a FliJ/Spa13 homolog, and with PcrD (FlhA/MxiA), to hinder fast PMF-dependent activity of the EA prior to activation signal reception, which then requires a rapid secretion burst [31].

Here, we sought to validate the role of PrcD/FlhA homolog MxiA in regulation of substrate selection and the role of the homologs of proteins known to interact with it in the *Pseudomonas* vT3SS and the *Salmonella* fT3SS, MxiC, IpaD and Spa13 in *Shigella*, in controlling the secretion rate. Using a genetic screen, we identified two amino acids located on the surface of MxiA’s cytoplasmic region (MxiA_C_) which, when mutated, upregulate late effector expression and secretion. We find these do not affect PMF-utilization during export but abolish the interaction of MxiA_C_ with MxiC in a 2-hybrid assay. Using this assay we also mapped the site of interaction with MxiA_C_ to the extreme C-terminus of MxiC. Finally we show that lack of MxiC and IpaD renders T3S in *Shigella* increasingly dependent on the ΔpH, rather than on the ΔΨ component of the PMF, suggesting these proteins also regulate a switch in secretion mode.

## Methods

### Bacterial strains and cell culture

All bacterial strains and plasmids used in this study are listed in Table 1. *S*. *flexneri* strains were maintained and selected on CR agar plates [47] and grown at 37°C in trypticase soy broth (TCSB; Becton Dickinson) supplemented with antibiotics when necessary (100 μg of ampicillin ml^-1^, 50 μg of kanamycin ml^-1^, 20 μg of chloramphenicol ml^-1^; Sigma). *E*. *coli* strains were maintained and selected on LB agar plates supplemented with antibiotics when necessary as for *S*. *flexneri*.

**Table 1:** *Shigella flexneri* and *E*. *coli* strains used in this study.

Strain	Genotype (strain; plasmid)	Reference
*Shigella flexneri*		
WT	Wild-type M90T, serotype 5a	[48]
WT/pHluorin	WT; pYVM007	This study and [49]
*ΔipaD*	SF622	[50]
*ΔmxiC*	WT with deletion of *mxiC*	[11]
*ΔmxiA*	WT with deletion of *mxiA*	This study
*ΔmxiA/mxiA*	*ΔmxiA*; pWSK29 *mxiA*	This study
*ΔmxiA/mxiA*_N373D_	*ΔmxiA*; pWSK29 *mxiA*_N373D_	This study
*ΔmxiA/mxiA*_Q608R_	*ΔmxiA*; pWSK29 *mxiA*_Q608R_	This study
*ΔmxiA/mxiA*_I674V_	*ΔmxiA*; pWSK29 *mxiA*_I674V_	This study
*E*. *coli* KS1 for two-hybrid assay (only fused genes are shown in the plasmid)		
Negative control	pRA02 pRA03 (pRA02 and pRA03 contain RNAP α subunit and λ cI, respectively)	[51]
Positive control	pRA02 *uvrA1*-252 pRA03 *mfd1*-219b	[51]
MxiC and MxiA_C_ interaction		
MxiC:MxiA_C318-686_	pRA02 *mxiC* pRA03 *mxiA*_C318-686_	This study
MxiC:MxiA_C318-686 N373D_	pRA02 *mxiC* pRA03 *mxiA*_C318-686 N373D_	This study
MxiC:MxiA_C318-686 Q608R_	pRA02 *mxiC* pRA03 *mxiA*_C318-686 Q608R_	This study
MxiC:MxiA_C318-686 I674V_	pRA02 *mxiC* pRA03 *mxiA*_C318-686 I674V_	This study
MxiC:MxiA_C356-686_	pRA02 *mxiC* pRA03 *mxiA*_C356-686_	This study
MxiC:MxiA_C356-686 N373D_	pRA02 *mxiC* pRA03 *mxiA*_C356-686 N373D_	This study
MxiC:MxiA_C356-686 Q608R_	pRA02 *mxiC* pRA03 *mxiA*_C356-686 Q608R_	This study
MxiC:MxiA_C356-686 I674V_	pRA02 *mxiC* pRA03 *mxiA*_C356-686 I674V_	This study
MxiC ΔC4:MxiA_C356-686_	pRA02 *mxiC* ΔC4 pRA03 *mxiA*_C356-686_	This study
MxiC ΔC9:MxiA_C356-686_	pRA02 *mxiC* ΔC9 pRA03 *mxiA*_C356-686_	This study
MxiC ΔC14:MxiA_C356-686_	pRA02 *mxiC* ΔC14 pRA03 *mxiA*_C356-686_	This study
MxiA_C356-686_:MxiC	pRA02 *mxiA*_C356-686_ pRA03 *mxiC*	This study
IpaD and MxiA_C_ interaction		
IpaD:MxiA_C318-686_	pRA02 *ipaD* pRA03 *mxiA*_C318-686_	This study
IpaD:MxiA_C318-686 N373D_	pRA02 *ipaD* pRA03 *mxiA*_C318-686 N373D_	This study
IpaD:MxiA_C318-686 Q608R_	pRA02 *ipaD* pRA03 *mxiA*_C318-686 Q608R_	This study
IpaD:MxiA_C318-686 I674V_	pRA02 *ipaD* pRA03 *mxiA*_C318-686 I674V_	This study
IpaD:MxiA_C356-686_	pRA02 *ipaD* pRA03 *mxiA*_C356-686_	This study
MxiA_C356-686_:IpaD	pRA02 *mxiA*_C356-686_ pRA03 *ipaD*	This study
Spa13 and MxiA_C_/IpaD/MxiC interaction		
Spa13:MxiA_C318-686_	pRA02 *spa13* pRA03 *mxiA*_C318-686_	This study
Spa13:MxiA_C318-686 N373D_	pRA02 *spa13* pRA03 *mxiA*_C318-686 N373D_	This study
Spa13:MxiA_C318-686 Q608R_	pRA02 *spa13* pRA03 *mxiA*_C318-686 Q608R_	This study
Spa13:MxiA_C318-686 I674V_	pRA02 *spa13* pRA03 *mxiA*_C318-686 I674V_	This study
Spa13:MxiA_C356-686_	pRA02 *spa13* pRA03 *mxiA*_C356-686_	This study
Spa13:MxiA_C356-686 N373D_	pRA02 *spa13* pRA03 *mxiA*_C356-686 N373D_	This study
Spa13:MxiA_C356-686 Q608R_	pRA02 *spa13* pRA03 *mxiA*_C356-686 Q608R_	This study
Spa13:MxiA_C356-686 I674V_	pRA02 *spa13* pRA03 *mxiA*_C356-686 I674V_	This study
Spa13:IpaD	pRA02 *spa13* pRA03 *ipaD*	This study
Spa13:MxiC	pRA02 *spa13* pRA03 *mxiC*	This study
Additional controls		
-:MxiA_C318-686_	pRA02 pRA03 *mxiA*_C318-686_	This study
-:MxiA_C318-686 N373D_	pRA02 pRA03 *mxiA*_C318-686 N373D_	This study
-:MxiA_C318-686 Q608R_	pRA02 pRA03 *mxiA*_C318-686 Q608R_	This study
-:MxiA_C318-686 I674V_	pRA02 pRA03 *mxiA*_C318-686 I674V_	This study
MxiA_C318-686_:MxiA_C318-686_	pRA02 *mxiA*_C318-686_ pRA03 *mxiA*_C318-686_	This study
MxiA_C318-686 N373D_:MxiA_C318-686 N373D_	pRA02 *mxiA*_C318-686 N373D_ pRA03 *mxiA*_C318-686 N373D_	This study
MxiA_C318-686 Q608R_:MxiA_C318-686 Q608R_	pRA02 *mxiA*_C318-686 Q608R_ pRA03 *mxiA*_C318-686 Q608R_	This study
MxiA_C318-686 I674V_:MxiA_C318-686 I674V_	pRA02 *mxiA*_C318-686 I674V_ pRA03 *mxiA*_C318-686 I674V_	This study
-:MxiA_C356-686_	pRA02 pRA03 *mxiA*_C356-686_	This study
MxiA_C356-686_:-	pRA02 *mxiA*_C356-686_ pRA03	This study
MxiA_C356-686_:MxiA_C356-686_	pRA02 *mxiA*_C356-686_ pRA03 *mxiA*_C356-686_	This study
Spa13:-	pRA02 *spa13* pRA03	This study
IpaD:IpaD	pRA02 *ipaD* pRA03 *ipaD*	This study
MxiC:MxiC	pRA02 *mxiC* pRA03 *mxiC*	This study

### Knockout and complementation of *mxiA*

In-frame deletion of inner membrane export apparatus protein MxiA was carried out by using the λ Red system [52]. A kanamycin resistance cassette was amplified from plasmid pKD4 using primers *mxiA*_KO_kanF and *mxiA*_KO_kanR and electroporated into *S*. *flexneri* wild-type carrying the Red recombinase to replace *mxiA*, giving rise to *ΔmxiA*, which was verified via sequencing primers *mxiA*_KO_ver_F and *mxiA*_KO_ver_R. Complementation was achieved by *in trans* expression of *mxiA* in the low copy plasmid pWSK29 [53], giving rise *ΔmxiA/mxiA*. *mxiA* mutants were obtained via in-fusion cloning (Clontech) using template pWSK29 *mxiA* and corresponding primers, such as MxiA_N373D-For and MxiA_N373D-Rev, as listed in Table 2. All constructs were verified by DNA sequencing (Eurofins).

**Table 2:** Primer sequences used in this study.

Primer	Sequence
Primers to knock out *mxiA* and verification of *ΔmxiA*	
*mxiA*_KO_kanF	5’-GTGCATACAAGAAAGAGCTTTCTAGATAACAGGAGATAAAAGTGATCCAGTCTTTTGTGTAGGCTGGAGCTGCTTC-3’
*mxiA*_KO_kanR	5’-TAACTAATTGAACTAAATTAATGTTACTCATATTTAAACCTCACTAAATAGTCTTTAACATATGAATATCCTCCTTAG-3’
*mxiA*_KO_Ver_F	5’-GCCAGTCATGAGGATTCTGTAG-3’
*mxiA*_KO_Ver_R	5’- TGGTAATCGCTGAATGGCTG-3’
Primers for PCR mutagenesis of *mxiA*	
mxiA_XbaI_RBS_F	AGTCTCTAGAAGATAACAGGAGATAAAAGTGATC
mxiA_EcoRI_R	AGTCGAATTCTAAATAGTCTTTAATACAT
Primers to make fusion proteins into pRA02 and pRA03	
IpaD_NheI_For	5’-GCGCGCTAGCAATGAATATAACAACTCTGACTAATAG-3’
IpaD_KpnI_Rev	5’-GCGCGGTACCTCAGAAATGGAGAAAAAGTTTATC-3’
MxiA_C318__XbaI_3G_For	5’-AGTCTCTAGAaggtggaggGGTCGTAGAAAAAGAAAAAAG-3’
MxiA_C356__XbaI_3G_For	5’-AGTCTCTAGAaggtggaggTATTAGTTCAGAAACCGTTC-3’
MxiA_KpnI_Rev	5’-AGTCGGTACCCTAAATAGTCTTTAATACA-3’
MxiC_NheI_For	5’-GCGCGCTAGCAGAGCTCATGCTTGATGTTAAAAATACAG-3’
MxiC_KpnI_Ror	5’-GCGCGGTACCGGATCCTTATCTAGAAAGCTCTTTCTTG-3’
Spa13_XbaI_3G_For	5’-AGTCTCTAGAaggtggaggGAAACAATTAGATAAGG-3’
Spa13_KpnI_Rev	5’-AGTCGGTACCTTATCTAATGCCATACTTC-3’
Primers for in-fusion cloning	
MxiA_N373D-For	5’-GAAAATAAGATAgATGCAAATG-3’
MxiA_N373D-Rev	5’-GCATcTATCTTATTTTCGGC-3’
MxiA_Q608R-For	5’-AGGGATAAGGCgAACCTCTG-3’
MxiA_Q608R-Rev	5’-GGTTcGCCTTATCCCTTTTC-3’
MxiA_I674V-For	5’-CGTATGCTGAGgTTGATGAAG-3’
MxiA_I674V-Rev	5’-CAAcCTCAGCATACGATATAACG-3’
MxiC_IF_For	5’-TAAGGATCCGGTACCCTAGAG-3’
MxiC_IF_delC4_Rev	5’-GGTACCGGATCCTTATTTCTTGTATG-3’
MxiC_IF_delC9_Rev	5’-GGTACCGGATCCTTATGTGACAAG-3’
MxiC_IF_delC14_Rev	5’-GGTACCGGATCCTTATAGAATATTG-3’

### MxiA mutant library screening

For reporter plasmid construction *mxiA*, followed by two transcription terminators, was cloned upstream of the reporter gene *cepH*, which encodes resistance to cephalosporins [54]. A 1485 bp DNA sequence (Figure A in S1 File; [17, 55]), modified from plasmid pQF50 and encoding two transcription terminators, the minimal promoter of *ipaH9*.8, and the *cepH* gene, flanked by *EcoR*I and *Xho*I restriction sites was synthesized by Eurofins and cloned downstream of *mxiA* in pWSK29 [53]. The resulting plasmid complemented *ΔmxiA* and the complemented strain was sensitive to 60–120 ng/ml cefotaxime (Sigma) on plates containing CR. *mxiA* was then amplified by error-prone PCR with 2.5–5 mM Mg^++^ and Taq DNA Polymerase (New England BioLabs) using primers *mxiA*_XbaI_RBS_F and *mxiA*_EcoRI_R, cloned into the reporter plasmid, transformed into DH5α to generate a library of MxiA mutants. The size of the libraries was estimated by plating a portion of the transformation mix on LB Ampicillin agar plates and the mutation rate was evaluated as 70% of sequences carrying one or more non-silent single base pair substitutions (predominantly to As and Ts) by randomly sequencing 10 transformants. Based on PEDEL, Programme for Estimating Diversity in Error-prone PCR Libraries (http://guinevere.otago.ac.nz/cgi-bin/aef/pedel.stats.pl), if we assume the mean number of point mutations per sequence is 0.7, 54,000 transformants are required to cover 95% of single point mutations in MxiA. The MxiA mutant libraries were transformed into *ΔmxiA* and screened on plates containing 60 ng/ml cefotaxime without CR and 120 ng/ml cefotaxime with CR, respectively.

### PMF inhibition assays

To test the role of the **Δ**Ψ and **Δ**pH in Ipa protein**s** export, we used carbonyl cyanide *m*-chlorophenyl hydrazone (CCCP, Sigma) as previously described [29, 31]. Briefly, cells at mid-log growth stage were washed twice in TCSB medium (pH ~7.3) containing 120 mM Tris (pH 7.0), and resuspended in TCSB containing either 0.5% DMSO or CCCP at concentrations up to 40 μM at 37°C for 30 min. To test the role of the **ΔΨ** alone, we used valinomycin (Sigma) as previously described [29, 31]: cells were washed further in TCSB containing 150 mM KCl after Tris treatment, then resuspended in TCSB containing 150 mM KCl supplemented with either 0.5% DMSO or valinomycin at concentrations up to 40 μM at 37°C for 30 min. To test the role of the **Δ**pH alone, cells were washed twice in 200 mM sodium phosphate buffer, pH 5.85, then resuspended in the same buffer containing potassium benzoate (Sigma) [56] at concentrations up to 40 mM at 37°C for 30 min. Both cells and supernatants were collected and analyzed by immunoblotting using the antibodies indicated in the figures.

### Intracellular pH measurement

*Shigella* WT was transformed with plasmid pYVM007, which encodes a pH-sensitive, ratiometric GFP derivative [49]. The bacteria were cultured overnight in TCSB medium with 100 μg/ml ampicillin, diluted 1:200 into fresh TCSB with 100 μg/ml ampicillin and 0.1 mM IPTG and grown to mid-logarithmic phase. The bacteria were then pelleted and resuspended in 200 mM sodium phosphate buffer with different concentrations of potassium benzoate or valinomycin with 150 mM KCl, and incubated for 30 minutes before measuring the fluorescence emission of pHluorin (520 nm emission) excited at 410 and 470 nm using a POLARstar Omega plate reader spectrophotometer (BMG). A standard curve was established by resuspending bacteria in 200 mM sodium phosphate buffer (pH 5.85, 6.34, 6.89, 7.41 and 7.91) in the presence of 40 mM potassium benzoate, and used to calculate the intracellular pH of the inhibitor-treated samples. Data is presented as ΔpH compared to the external pH of the buffer used.

### Change of membrane potential

The change in membrane potential of PMF inhibitor-treated *S*. *flexneri* was detected using potential sensitive fluorescent dye DiSC3(5) (Sigma) as previously described [31]. Briefly, the overnight WT bacteria were diluted 1:200 in fresh TCSB medium and grown to mid-logarithmic phase. Cells were pelleted, washed and resuspended in 5 mM HEPES buffer, pH 7.7, to an OD_600_ of 0.1 supplemented with 0.4 μM DiSC3(5) dye. Cells were incubated at room temperature for 20 minutes, at which point 100 mM KCl (final concentration) was added. The cells were then treated with potassium benzoate or valinomycin and incubated for an additional 30 minutes. Increase in DiSC3(5) fluorescence due to the disruption of the membrane potential gradient (ΔΨ) was detected using a POLARstar Omega plate reader spectrophotometer (BMG, excitation 620 nm, emission 670 nm). Data is presented as fold change in fluorescence intensity relative to wild-type bacteria without inhibitor treatment.

### Analysis of Ipas secretion and protein expression

The secretion of Ipa proteins after Congo red induction was carried our as previously described [11]. The total level of protein expression was revealed via western blot using monoclonal anti-IpaB H16 [57] and polyclonal anti-IpaH [58], anti-RNAP α subunit (gift from Prof. Akira Ishihama, Hosei University, Koganai, Tokyo) and anti-λ cI (gift from Prof. Ann Hochschild, Harvard Medical School, Boston). Goat anti-mouse DyLight 800 (Fisher Scientific) or goat anti-rabbit Alexa 680 (Invitrogen) conjugates were used as secondary antibodies. The membranes were then visualized using an Odyssey infrared imaging system (LI-COR Biosciences).

### Bacterial two-hybrid assay

The bacterial 2-hybrid assay used in this work is essentially that developed by Dove and Hochschild [59]. Reporter strain *E*.*coli* KS1, which carries a chromosomal *lacZ* gene under the control of a *lac* promoter *plac*O_R_2-62, can be activated by interactions between a protein fused to λ cI and a protein fused to the RNA polymerase (RNAP) α subunit. To generate the plasmids, genes to-be-fused were PCR amplified using primers listed in Table 2, double digested with *Kpn*I and *Xba*I (for *mxiA* and *spa13*) or *Nhe*I (for *ipaD* and *mxiC*), and then ligated into plasmids pRA02, which encodes RNAP α subunit and pRA03, which encodes λ cI [51], both double digested with *Kpn*I and *Xba*I (producing an overhang compatible with *Nhe*I). For genes with mutations to-be-fused, such as *mxiA*_*C356-686 N373D*_, in-fusion cloning (Clontech) was performed using either pRA02 or pRA03 *mxiA*_*C356-686 N373D*_ as PCR template via primers MxiA_N373D-For and MxiA_N373D-Rev. All primers are listed in Table 2 and all constructs were verified by DNA sequencing (Eurofins). *E*.*coli* KS1 cells were transformed with the combinations of pRA02 and pRA03 derivatives listed in Table 1. An overnight culture was diluted and grown to mid-log phase (A600 0.3~0.5) in LB broth supplemented with IPTG (1 mM) and antibiotics. β-galactosidase activity was assayed as described by Miller [60].

## Results

### MxiA double mutant displays increased expression of the IpaH family of late effectors

To identify mutation(s) involved in intracellular activation of the *Shigella* T3SS, we constructed a reporter plasmid where the cefotaxime resistance gene *cepH* [54] is under the control of the minimal promoter of the late effector gene *ipaH9*.*8* [17]. The expression of *ipaH9*.*8* is only turned on upon T3SS activation. Hence, we can select for mutants leading to *ipaH9*.*8* expression via *pipaH9*.*8 cepH*-dependent generation of resistance to cefotaxime. We used this plasmid, where *mxiA* followed by transcription terminators was cloned upstream of *pipaH9*.*8 cepH*, to screen for *mxiA* mutants leading to cefotaxime resistance in a *ΔmxiA* background. As MxiA is an essential component of the T3SS CEA, only gain-of-function *mxiA* mutants can be returned by the screen since those that disrupt function will not answer the selection. Amongst an estimated 5000 transformants, which cover about 20% of potential single mutation of MxiA in our screening, we identified one cefotaxime-resistant transformant, which bears two mutations: N373D and I674V. The need to use a low copy plasmid for complementation and as a reporter limited the transformation and hence screening efficiency, making it impractical to seek to saturate the screen. The mutant showed 2 to 3 fold increased expression of IpaH proteins compared to WT and *ΔmxiA*/*mxiA* (Figure B, panel a in S1 File). To further understand the role of each of these mutations, we constructed *ΔmxiA*/*mxiA*_N373D_ and *ΔmxiA*/*mxiA*_I674V_. For comparison, we also made *ΔmxiA*/*mxiA*_Q608R_ based on a recent publication [31], which identified PcrD_Q626R_, which is homologous to MxiA_Q608R_, as resulting in activation of effector secretion under normally repressive conditions in *Pseudomonas aeruginosa*. The location of the three mutations is shown in Fig 1B and 1C: they are all on the surface of the globular C-terminal domain of the protein. I674 lies quite close to Q608 on the bottom (cytoplasm-facing) part of the inner face while N373 lies in an unstructured loop on the upper (membrane-facing) part of the outer face of the putative nonameric ring.

### MxiA_I674V_ behaves similarly to WT but secretes more late effector protein IpaH relative to early effector IpaB

**Fig 2:**
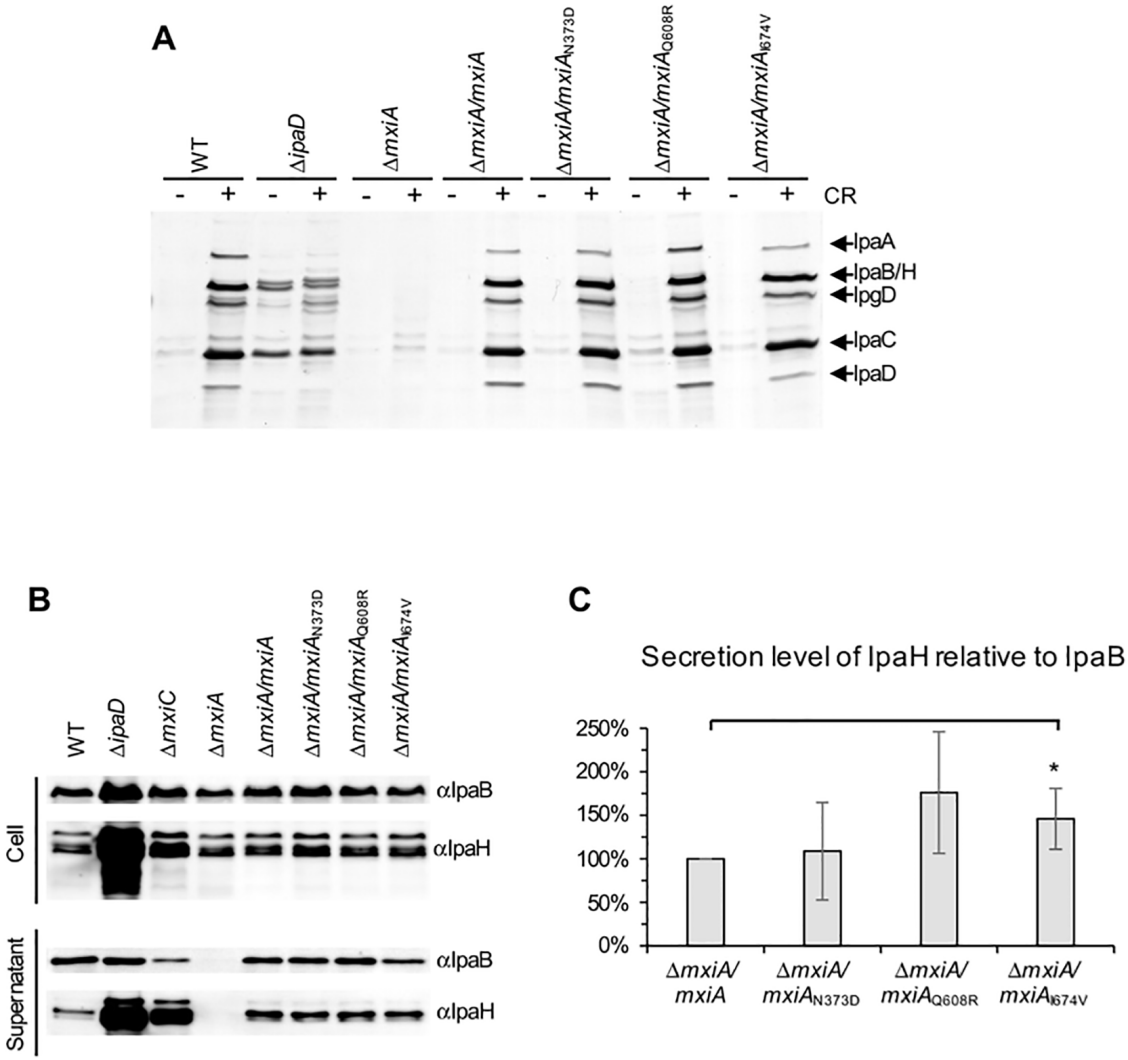
MxiA_I674V_ mutant behaves similarly to WT but secretes more late effector protein IpaH relative to early effector IpaB. (A) Induced secretion of Ipa proteins after the addition (+) or in absence (-) of Congo red (CR) was analysed by silver staining. The positions of the major Ipa proteins detected are indicated on the right side. (B) Overnight total culture (top) and supernatant (bottom) were analysed by immunoblotting using antibodies against translocator IpaB or later effector IpaH as indicated. Bacterial numbers were normalized by OD_600_ and the data shown here are representative of three independent experiments giving similar results. (C) Secretion level of IpaH relative to the secretion level of IpaB in *mxiA* mutants adjusted to complemented strain whose ratio was set as 100%. Statistical analysis was performed using R (https://www.R-project.org/). The statistical significance of the differences compared with the control is noted based on four experiments: *p = 0.021, Wilcoxon test; p = 0.078, Welch two samples t-test.

To understand the effect of these MxiA mutations, we carried out functional assays of T3SS activity, including Congo red (CR) induction and overnight leakage. “Induction” describes the burst of Ipa protein secretion upon host cell sensing [61] or addition of the artificial inducer CR [62]. “Overnight leakage” is a slow, low-level Ipa protein secretion prior to host cell contact whereby around 5% of Ipa proteins are secreted [63]. With respect to CR induction (Fig 2A), *ΔmxiA*/*mxiA*_N373D_, *ΔmxiA*/*mxiA*_Q608R_ and *ΔmxiA*/*mxiA*_I674V_ all behaved like the wild-type and *ΔmxiA*/*mxiA*. In contrast, the *ΔmxiA* was uninducible by CR due to the lack of a functional T3S apparatus, and *ΔipaD* was insensitive to CR because it lacks a tip complex [5] and is a known constitutive secretor [64]. In comparison to wild-type and complemented strains, all mutants showed similar expression levels of later effectors IpaH relative to the early effector IpaB (Fig 2B and Figure C, panel a in S1 File). No differences were observed between any mutants and the complemented strain for the secretion level of IpaH relative to its expression nor for the secretion level of IpaB relative to its expression (Figure C, panels b & c in S1 File). However, MxiAI674V secreted more later effectors IpaH relative to the early effector IpaB compared with the complemented strain, although the statistical significance of the difference observed was marginal (Fig 2C). This is similar, if less drastic as might be expected from the subtler nature of the mutations involved, to what was previously reported for *ΔmxiC* (Fig 2C; [11]). Taken together, these data suggest that at least amino acid I674 within MxiA is involved in regulating the secretion switch between early and later effectors.

### The MxiAI674V mutant and WT respond similarly to PMF inhibitors, but *ΔmxiC* and *ΔipaD* respond differently

Evidence has been provided that protein export via flagellar and virulence T3SSs relies partly on the PMF across the bacterial inner membrane as an energy source, which may be utilized by MxiA homologs [29–32, 65, 66]. If the *mxiA* mutants we isolated allow the *Shigella* T3SS to use the PMF more efficiently, then these mutations should render the export process more resistant to the collapse of the PMF. Therefore, we tested the ability of our *mxiA*_I674V_ mutant to modulate the sensitivity of translocator export to the addition of PMF inhibitors, as compared to WT, *ΔmxiC* and *ΔipaD*.

**Fig 3:**
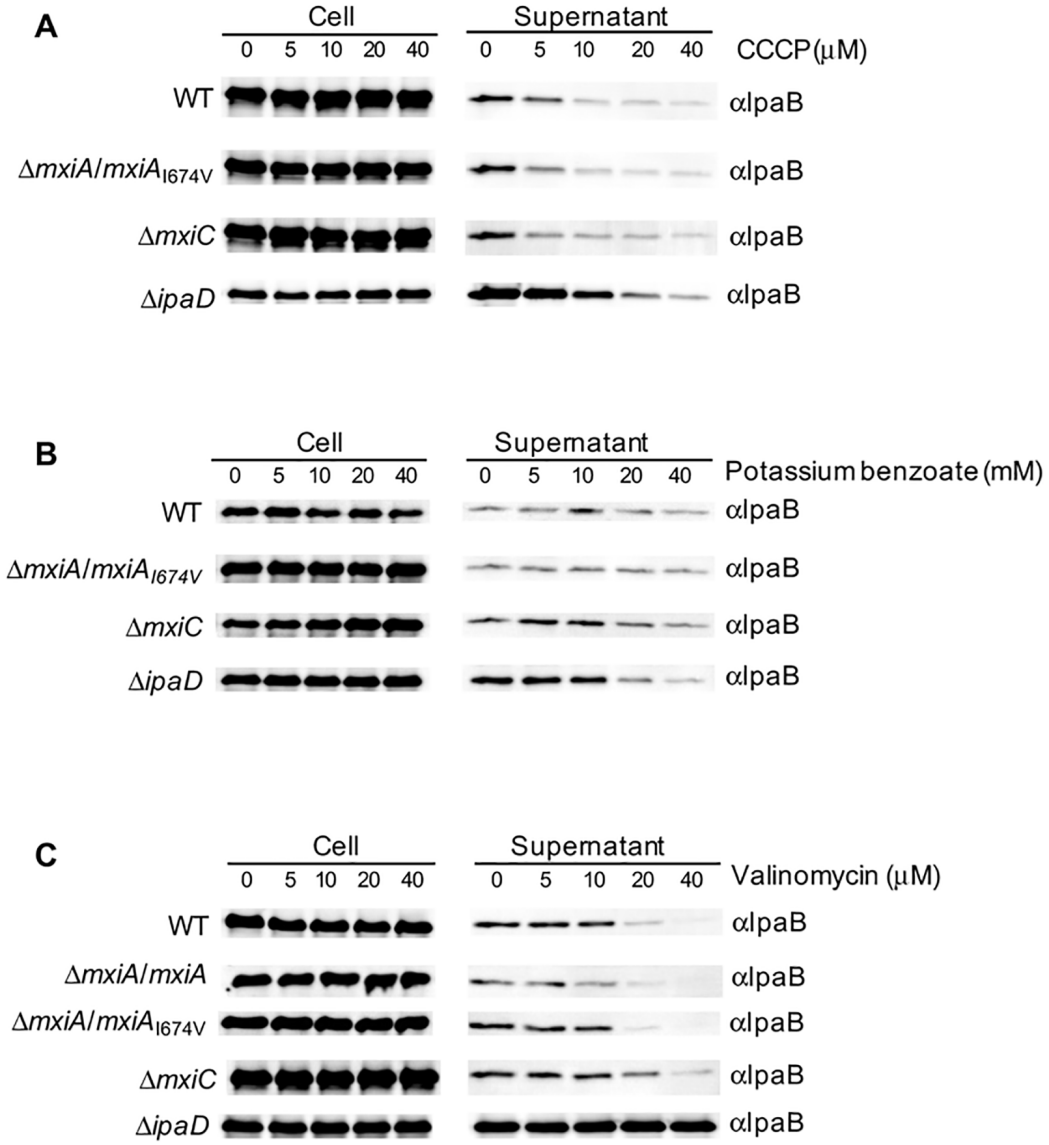
MxiA mutants and WT respond similarly to PMF inhibitors, but *ΔmxiC* and *ΔipaD* respond differently. Bacteria were treated with CCCP in TCSB medium (A), or potassium benzoate in 200 mM sodium phosphate buffer, pH 5.85 (B), or valinomycin in the presence of 150 mM KCl in TCSB medium (C) and T3SS-mediated log-phase “leakage” was analysed. The cell (left) and supernatant (right) fractions were analysed by immunoblotting using antibodies against translocator IpaB. Bacterial numbers were normalized by OD_600_ and the data shown here are representative of 2 or 3 independent experiments giving similar results.

The PMF consists of two components, an electric potential difference between the periplasmic and cytoplasmic faces of the membrane (ΔΨ) and a proton concentration difference (ΔpH). CCCP functions as an ionophore to discharge both ΔΨ and ΔpH [29], valinomycin shuttles potassium into the cell, thereby balancing the difference in charge and collapsing the ΔΨ [29], while benzoic acid is a membrane-permeant weak acid which can enter the cell and collapse the ΔpH of the PMF when added in a low external pH environment [67]. In TCSB medium, IpaB secretion was strongly inhibited by 10 μM CCCP in all strains (Fig 3A and Figure D in S1 File). Potassium benzoate, but not valinomycin/KCl, disrupts the ΔpH in *Shigella* at external pH 5.85 (Figure F, panels a & b in S1 File), but not at external pH above 7 (not shown) or in TCSB medium due the changing external pH therein (Figure G in S1 File). While 20 mM potassium benzoate dramatically inhibited IpaB secretion in Δ*ipaD* and to a lesser extent in Δ*mxiC*, it had no effect on the secretion of IpaB in WT or *mxiA*_I674V_ (Fig 3B and Figure E in S1 File). Valinomycin/KCl, but not potassium benzoate, collapses the ΔΨ (Figure F, panels c & d in S1 File). While valinomycin/KCl inhibits the secretion of IpaB in both wild-type and *mxiA* mutants at 20 μM, *ΔipaD* is totally resistant to valinomycin up to 40 μM and *ΔmxiC* is intermediately-resistant to the effect of this drug (Fig 3C and Figure H in S1 File). Thus, WT and our *mxiA* mutants use the PMF similarly, and primarily the ΔΨ, to secrete translocator IpaB. Interestingly, however, lack of either of two regulators, MxiC or IpaD, the homologs of which were recently shown to interact with the MxiA homolog PcrD in *Pseudomonas* [31], renders IpaB secretion increasingly dependent on the ΔpH, but independent of the ΔΨ.

### The *mxiA* mutants no longer interact with MxiC

**Fig 4:**
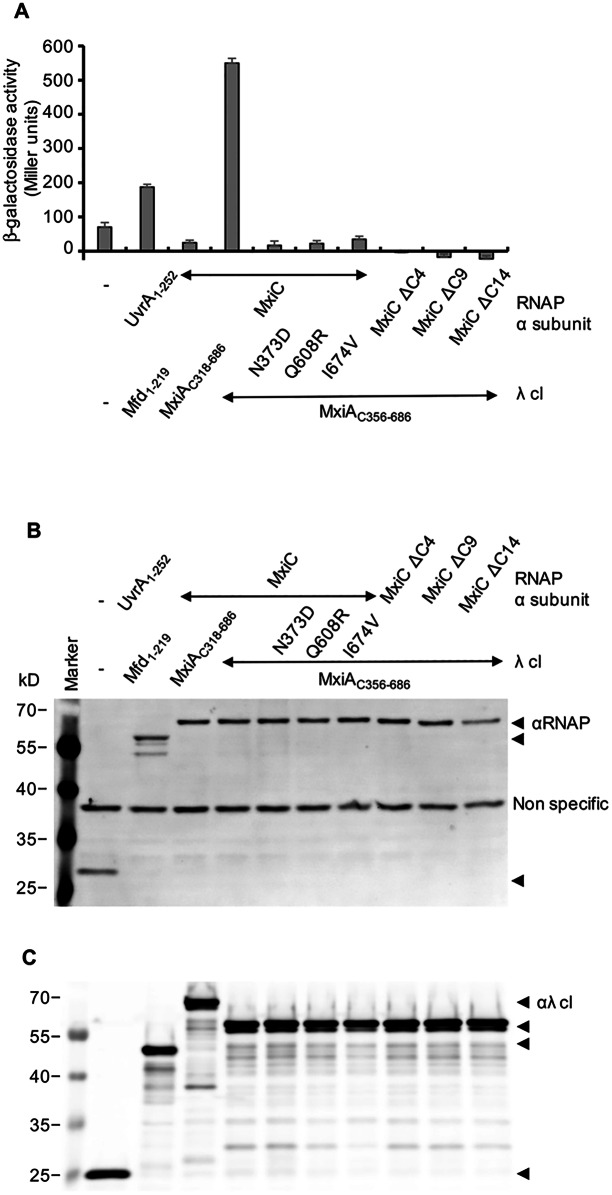
MxiA mutants no longer interact with MxiC in a bacterial two-hybrid assay. MxiC and its C-terminal deletion derivatives were fused to RNAP α subunit in plasmid pRA02, and MxiA_C_ and its mutants were fused to λ cI in plasmid pRA03. Interaction between the fusion proteins recruits RNAP to the lacZ reporter construct, and β-galactosidase activity reflects the strength of the interaction (A). ‘‘-” indicates the expression of RNAP α subunit or λ cI only, UvrA1-252 interact with Mfd1-219b [51] and was used as a positive control. Production of the indicated RNAP α subunit and λ cI-fusion proteins was analysed by immunoblotting using antibodies against RNAP α subunit (B) and λ cI (C). Bacterial numbers were normalized by OD_600_ and the data shown here are representative of 2 independent experiments giving similar results.

To determine the reason(s) why our MxiA mutants secrete IpaH proteins more efficiently, we set-up two-hybrid assays to test if they interact with their potential partners differently. PcrD, a homologue of MxiA, co-precipitates with Pcr1 [31], which corresponds to C-terminal domain 3 of MxiC. To test the interaction between the C-terminus of MxiA (MxiA_C_) and MxiC, MxiA_C_ was fused to λ cI, and MxiC was fused to RNAP α subunit as described in the Materials and Methods. Interaction-dependent recruitment of RNA polymerase to the promoter leads to the expression of β-galactosidase, of which the activity was monitored. MxiC interacts strongly with MxiA_C356-686_, but not when it is fused to λ cI and MxiA_C_ to RNAP α subunit It also does not interact with MxiA_C318-686_ in either fusion configuration (Fig 4A and not shown). The latter constructs encode the whole cytoplasmic domain, including what is probably an extended linker region linking it to its transmembrane region [36]. Therefore, the linker region may regulate the MxiC-MxiA interaction assessed here. Yet, lack of binding in the presence of a more complete MxiA construct is a negative result and what we remove is a flexible linker, the conformation of which can not be assumed to be native in the two-hybrid fusion protein. However, although all constructs showed similar expression levels of fusion proteins (Fig 4B), RNAP α subunit-MxiC does not interact with λ cI-MxiA_C356-686_ bearing mutations N373D, Q608R or I674V. PcrD_Q626R_ showed reduced interaction with Pcr1 [31]. Thus our data indicate that mutations MxiA_Q608R_ or nearby MxiA_I674V_ lead to a similar defect, and one that is caused also by the more distantly located mutation MxiA_N373D_. In parallel, we also sought to map the site of interaction of MxiC on MxiA_C_ using two-hybrid assays. Consistent with the fact that Pcr1 is homologous the C-terminal quarter of MxiC, although all constructs are expressed (Fig 4B), MxiC with C-terminal deletions of its last four to fourteen amino acids lost the ability to bind MxiA_C356-686_.

The work of Lee & Rietsch in *Pseudomonas* [31] showed that PscO, the homologue of *Shigella* Spa13, and PcrG, the homologue of the N-terminus of IpaD, interact with PcrD, the MxiA homologue. Furthermore, they found that PcrG and Pcr1 interact. The interaction of PcrG & PcrD and PcrG & Pcr1 were shown to be direct, while Minamino et al. showed the FliJ, the Spa13 homolog, binds FlhA, the MxiA homolog directly [32, 37]. Therefore, we further checked for interactions between MxiA_C_ (both with and without linker region and as RNAP α subunit or λ cI fusions, except in combination with the *spa13* fusion, where only RNAP α subunit-Spa13 and λ cI-MxiA_C_ could be obtained; Table 1), Spa13, MxiA_C_ and IpaD. However, no interaction was detected for any of these pairs (data not shown). In this work and previous work, we also tested for IpaD-MxiC interactions in this assay, also without success [23].

## Discussion

In this work, we demonstrate that the *Shigella* T3SS inner membrane protein MxiA plays a role in substrate selection. We also show that MxiC and IpaD, two cytoplasmic regulators already shown to play a role in secretion hierarchy control and to negatively regulate the secretion rate, also control the export apparatus’ mode of PMF utilization. Finally, we also show that the cytoplasmic region of MxiA interacts directly with MxiC and identify residues important for this interaction in both proteins.

Our *mxiA* mutants stimulate expression and secretion of some proteins of the IpaH late effector family, but not of the translocator IpaB. The *pcrD*_Q626R_ mutant upregulates expression of the ExoS effector, but its full secretion profile was not shown by Lee & Rietsch [31]. Interestingly, the *pcrG*_Δ30–40,Δ60–70_ and the *pscO*_G78E or E88K_ mutants they characterised have a different phenotype: they affect mainly the secretion rate, not its specificity [31]. Thus, the cytoplasmic complex formed around MxiA/PcrD is a major substrate selection point.

As expected from the genetic screen we used to isolate them, the mutations in MxiA that affect substrate selection are very mild and do not affect the other, essential function(s) of the protein in T3S. Unlike the partial loss-of-function *prcG* and *pscO* mutants described by Lee & Rietsch (2014), our MxiA point mutants also do not affect PMF utilisation by the T3SS. The latter was not tested by these authors but as we made a homologous mutant (MxiA_Q608R_) to the strongest one they found in PcrD, we assume this is also the case in *Pseudomonas*. Interestingly, however, each of our point mutants leads to complete loss of interaction with MxiC in our 2-hybrid assay but PcrD_Q626R_ leads only to a partial loss of interaction with Pcr1 in pull-downs [31]. This suggests other factors can stabilise this interaction *in vivo*, although we could not detect the ones we expected from the work of Lee & Rietsch (2014) in our 2-hybrid assays. This may be because of slight differences in the assays used or in the cloning of the fusion proteins. More interestingly, this may also be due to differences in how some of these proteins are found at steady state. Indeed, MxiC is equivalent to PopN and Pcr1, and this complex may a have different conformation [20] to that of MxiC [21], whereas IpaD is equivalent to PcrV and PcrG, meaning it may not have its relevant surfaces available for interaction with MxiC. Might such differences influence the fine-tuning of this regulatory cascade in each species?

On the other hand, lack of the MxiC extreme C-terminus which, according to the MxiC crystal structure [21], is unlikely to affect its folding dramatically and which causes loss of MxiA binding in the 2-hybrid assay, is a complete loss-of-function mutant in *Shigella* [23, 68]. While relatively conservative substitutions are likely less disruptive than short deletions, this suggests that the site targeted by the MxiC C-terminus on MxiA is important to formation of this regulatory complex. Although we have insufficient information to dock the MxiA_C_ and MxiC structures together, the location of our MxiA mutants suggest MxiC may wrap around the MxiA nonamer, from the outside to the inside of the ring.

How our MxiA mutations act to upregulate *ipaH* gene expression is not clear. Our mutants do not demonstrate detectably increased “leakage” (Figure B, panel b in S1 File). Therefore, this slight upregulation is unlikely to be occurring via the previously characterised MxiE transcriptional activator. This protein requires its co-activator, IpgC, the cytoplasmic chaperone of IpaB and IpaC translocators, to have been liberated from these by their secretion to interact with MxiE [58]. Interestingly, a *mxiC* mutant shows a 2-fold decrease in number of T3SSs/cell [11]. This suggests it is required as a positive regulator of T3SS gene expression. In addition, one component of the heterodimeric chaperone complex of the *Chlamydia* MxiC homolog CopN acts as an inhibitor of RNA polymerase by binding to its σ^66^ subunit, when not part of the CopN complex [69, 70]. Therefore, a role for the MxiA-MxiC interaction in regulating late effector transcription/translation cannot be excluded. However, that our MxiA mutations do mildly alter substrate selection is supported by the fact that all but one of the mutations isolated by Lee & Rietsch [31] and ourselves localise to MxiA/PcrD domain 4 (Fig 1B and 1C), the main domain involved in substrate selection in their flagellar homolog FlhA [44, 45].

In the *Pseudomonas* vT3SS, both components of the PMF were required to power translocator export [31]. In *Pseudomonas*, a partial defect in PcrG (the homolog of IpaD’s N-terminus) was shown to render their secretion of translocators more resistant to dissolution both of the ΔΨ and of the ΔpH, suggesting they can use these more efficiently [31]. But, in the *Salmonella* fT3SS, only the ΔΨ is required to support flagellar protein export in wild-type cells [65], as we see here for wild-type *Shigella* vT3SS and our MxiA mutants also. However, in a *Salmonella* fT3SS mutant lacking flagellar ATPase complex and carrying a bypass point mutation (P28T) in FlhB -the Spa40 homolog- which enhances protein export, Minamino et al. (2011) observed a dual requirement for ΔΨ and the ΔpH [32]. The latter is reminiscent of the requirements we see here for the *ΔmxiC* mutant, while the *ΔipaD* mutant, which has the most accelerated secretion rate, requires only the ΔpH. This suggests that the observation made by Minamino et al. also applies to circumstances where the ATPase complex is present and the IMEA is intact and that MxiC and IpaD negatively regulate the rate of protein export by controlling the mode in which MxiA utilizes the PMF. Thus, once again, our data suggest this functional and regulatory pathway is at least partially conserved, and hence of importance, amongst T3SSs. However, how MxiA_I674_ is altered in substrate secretion selection and how WT, Δ*mxiC* and Δ*ipaD* use the PMF differently need further investigation.

## Supporting Information

S1 File Supplementary Figures A to H. (PDF)
