# Supplementary material for: MxiA, MxiC and IpaD Regulate Substrate Selection and Secretion Mode in the T3SS of Shigella flexneri
Source: PLoS One. 2016 May 12;11(5):e0155141. doi: 10.1371/journal.pone.0155141 (PMC4865121; doi:10.1371/journal.pone.0155141)

SUPPLEMENTARY MATERIAL FOR

MxiA, MxiC and IpaD Regulate Substrate Selection and Secretion Mode in the T3SS of *Shigella flexneri*

Da-Kang Shen[1] and Ariel J. Blocker[2]

[1]School of Cellular & Molecular Medicine, Faculty of Biomedical Sciences, University of Bristol, BS8 1TD, Bristol, United Kingdom.

[2]Schools of Cellular & Molecular Medicine and Biochemistry, Faculty of Biomedical Sciences, University of Bristol, BS8 1TD, Bristol, United Kingdom.

FIG. A. Sequence of synthesized reporter construct

ter2-p*ipaH*9.8-*cepH* (1,485 bp)

GAATTCAAAAAACCGCACACCTTACTGGTGTGCGGTTTTTCGAGCCCGCCTAATGAGCGGGCTTTTTTTTCGAC
GCTCTCCCTTATGCGACTCCTGCATTAGGAAGCAGCCCAGTAGTAGGTTGAGGCCGTTGAGCACCGCCGCCGCAA
GGAATGGTGCATGCCTGCAGGCCTgGAGATCTCCATGGACGCGTGACGTCGACTCTtGAGGATCCCTGAAACAGT
ATCGTTTTTTTACAGCCAATTTTGTTTATCCTTATTATAATAAAAAAGTGCTGAAGTTCATTTCATGGAATGAAC
TTCAAAGCTTCTAGCTAGAGGGTATTAATAATGAAACAGACCAGATCCCTGCCACTGCTGGCCCTCGGCACCCTG
CTGCTTGCCCCGCTTTCCCTGGCGGCCCCCGTCGATCCGCTGAACGCTGTGGTGGATGACGCCATCCGCCCCATG
CTCAAGCAGCACAGGATCCCGGGCATGGCGGTCGCCGTGCTGAAGGGGGGGCAGGCCCACTACTTCAACTACGGG
CTGGCCGATATGGCGGCCGGGAAGAAGGTCAGCGAGCAGACCCTGTTCGAGATTGGCTCGGTCAGCAAAACCTAT
ACCGCCACCCTTGGTGCCTACGCCGTGGTCAAGGGGGGGATTGGGCTGGATGACAAGGTGAGCCGGCACGCGCCC
TGGCTCAAGGGCTCCGCCTTCGATGGCGTCACCATGGCGGAGCTCGCCACCTACAGCGCCGGCGGGCTGCCGCTG
CAATTCCCCGACGAGGTGGAATCGGTCGAACAAATGCAGTCTTACTATCGCCAGTGGACGCCGGCCTATCAGCCG
GGCAGCCATCGCCAGTACTCCAATCCCAGTATCGGTCTGTTCGGCCATCTGGCGGCGAGCAGCCTGCAGCAGCCA
TTTGCCCAGTTGATGGAGCAGACCCTGCTGCCCGGGCTCGGCTTGCATCACACTTATCTCGATGTGCCGGAAGCA
GCCATGGCGAGTTATGCCTATGGCTATTCGAAGGAGGACAAGCCTATCAGGGTCAATCCCGGCATGCTGGCCGAC
GAGGCCTACGGCATCAAGACCAGCTCGGCGGATCTGCTCGCCTTCGTGAAGGCCAATATCAGCGGGGTTGATGAC
AAGGCGTTGCAGCAGGCCATCTCCCTGACCCACCAAGGGCGCTACTCGGTCGGCGAGATGACCCAGGGGCTGGGC
TGGGAGAGTTACACCTATCCCGTCAGCGAGCAGACGCTGCTGGCGGGCAACTCCTCGGCGGTGATTTACAATGCC
AACCCGGTCAAACCTGTTGCCGCGTCCCAAGAGACGGGGGGGGCGCGACTCTACAACAAGACCGGCTCGACCAAC
GGCTTTGGTGCCTATGTGGCCTTCGTGCCGGCCAAAGGGATCGGCATCGTCATGCTGGCCAACCGCAACTACCCC
AACGAAGCCAGAATCAGCGCGGCCCATGCCATTTTGAGCCAGCTGGCCCCCTGA**CTCGAG**

The *ipaH*9.8 promoter sequence is underlined.
The sequences in red are two Rho-independent transcription terminators.
The beginning and the end of the sequence, in bold, are *EcoR*I and *Xho*I sites, mutated from the *Xho*I site and the *Xba*I sites in pQF50, respectively.

FIG. B. Strain Δ*mxiA*/*mxiA*$_{N373D\ I674V}$ expresses more late effector IpaH proteins compared to wild-type. (A) Overnight total cultures were analysed by immunoblotting using antibodies against late effector IpaH proteins. The strain with double mutations in *mxiA* was obtained during the screening as resistant to cefotaxime. The bands boxed in red were quantified using the software supplied for The Odyssey by LI-COR Biosciences. (B) The same cultures used in A had their supernatants analysed for 'leakage' via SDS-PAGE and Silver stain.

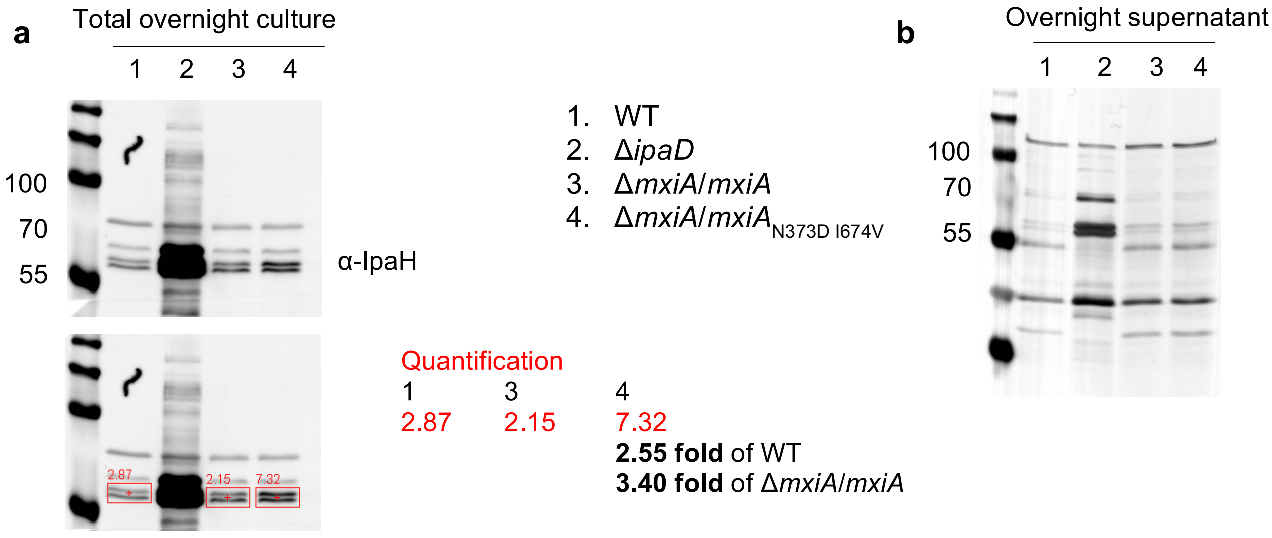

FIG. C. Relative expression and secretion level of IpaH and IpaB in *mxiA* mutants. (A) Expression level of IpaH relative to that of IpaB. (B) Ratio of the amount of IpaH secretion relative to its expression. (C) Ratio of the amount of IpaB secretion relative to its expression. All calculations were adjusted to the complemented strain Δ*mxiA*/*mxiA*, the value of which was set as 100%. Based on four experiments, no statistically significant differences were found for the mutants when compared with the complemented strain, using either the Wilcoxon test or the Welch two samples t-test.

a

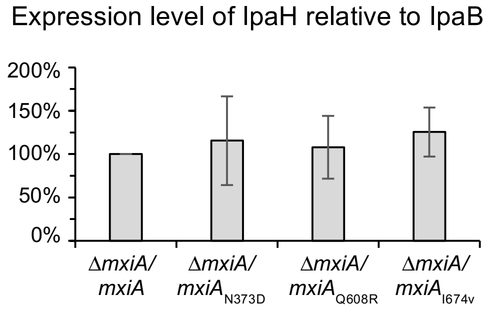

b

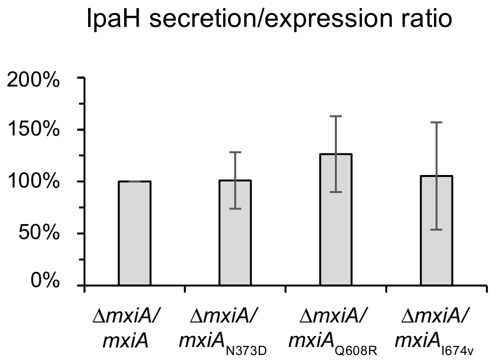

c

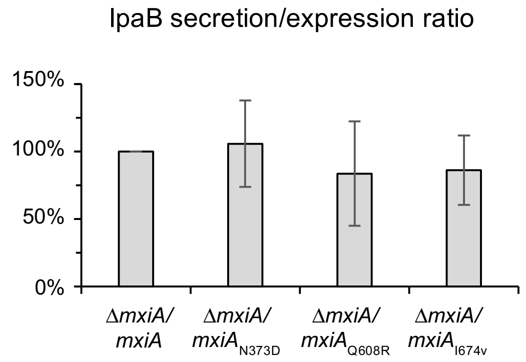

FIG. D. Effect of CCCP on T3S in *Shigella*. Bacteria were treated with CCCP in TCSB medium. The secretion level of IpaB in the absence of CCCP was set as 100% and the data shown were calculated from two experiments. One representative result is shown in Fig. 3A.

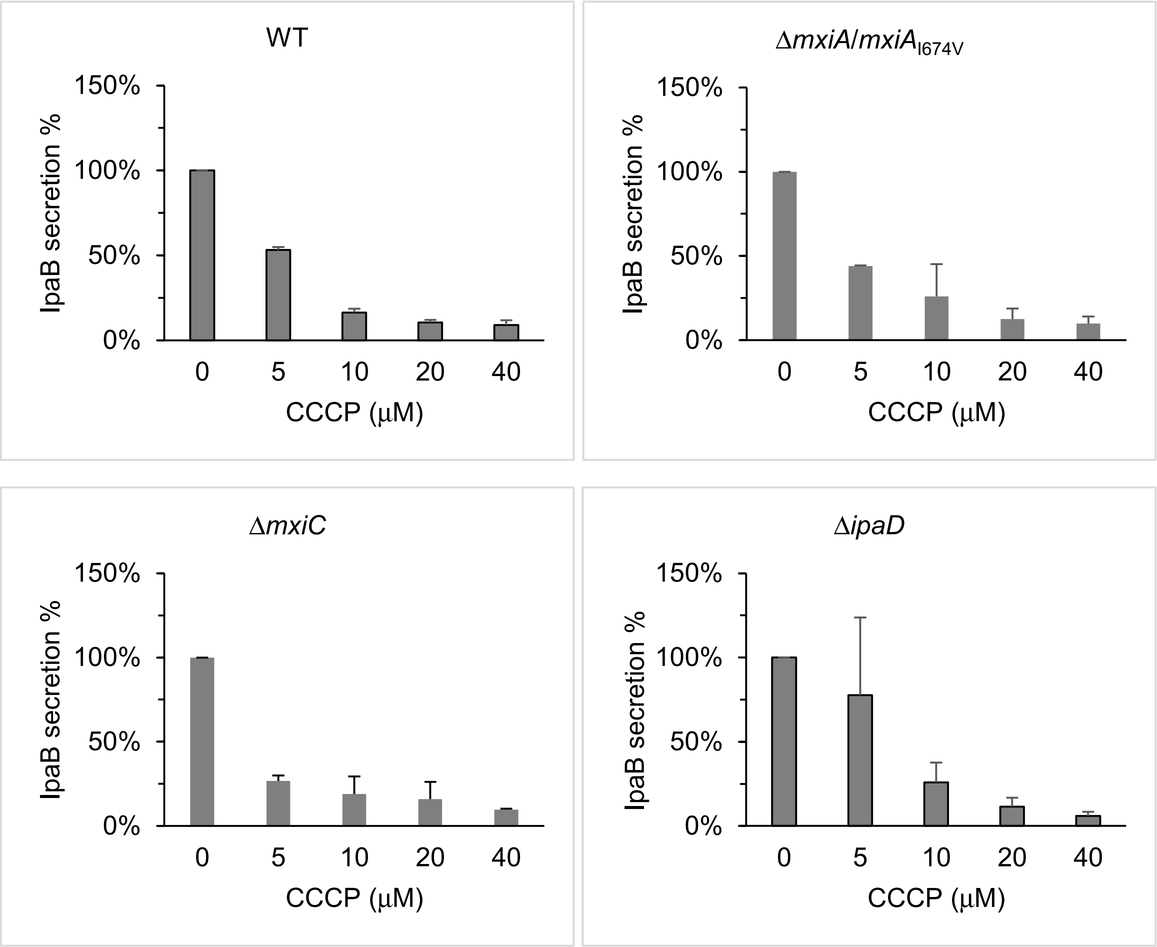

FIG. E. Effect of potassium benzoate on T3S in *Shigella*. Bacteria were treated with potassium benzoate in 200 mM sodium phosphate buffer, pH 5.85. The secretion level of IpaB in the absence of potassium benzoate was set as 100% and the data shown were calculated from three experiments. One representative result is shown in Fig. 3B.

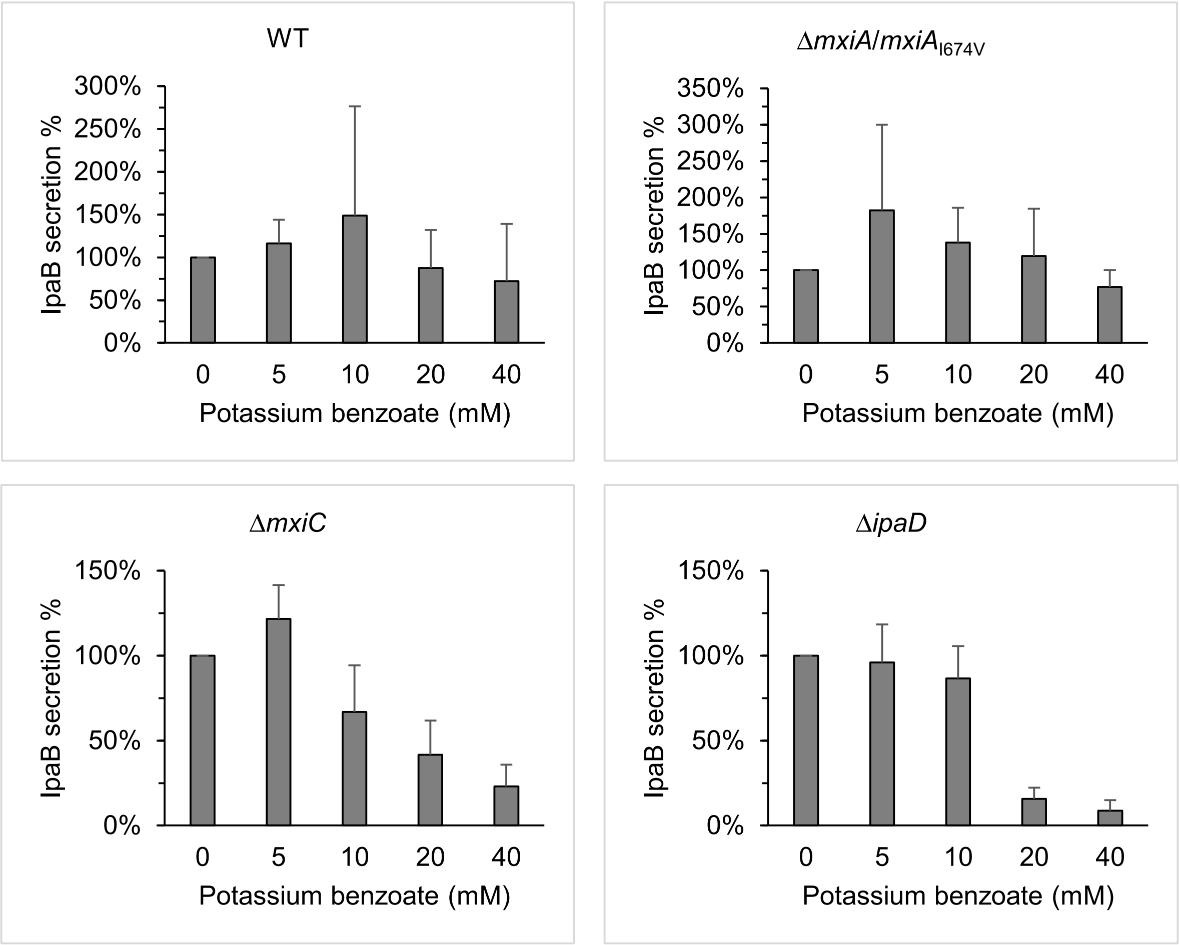

FIG. F. Measurement of ΔpH and membrane potential. To measure intracellular pH, *Shigella* WT (pYVM007), which produce the pH-sensitive ratiometric GFP derivative pHluorin, were washed and resuspended in 200 mM sodium phosphate buffer pH 5.85 with different concentrations of potassium benzoate (A) or valinomycin with 150 mM KCl (B). ΔpH is expressed as the difference between intracellular pH relative to external pH 5.85. To measure membrane potential, *Shigella* WT were washed and resuspended in 5 mM HEPES, pH 7.7 containing 100 mM KCl and 0. 4 μM dye DiSC3(5). Membrane potential is expressed as fold change in fluorescence in the presence of potassium benzoate (C) or valinomycin (D) relative to untreated WT cells. The data shown were calculated from two experiments.

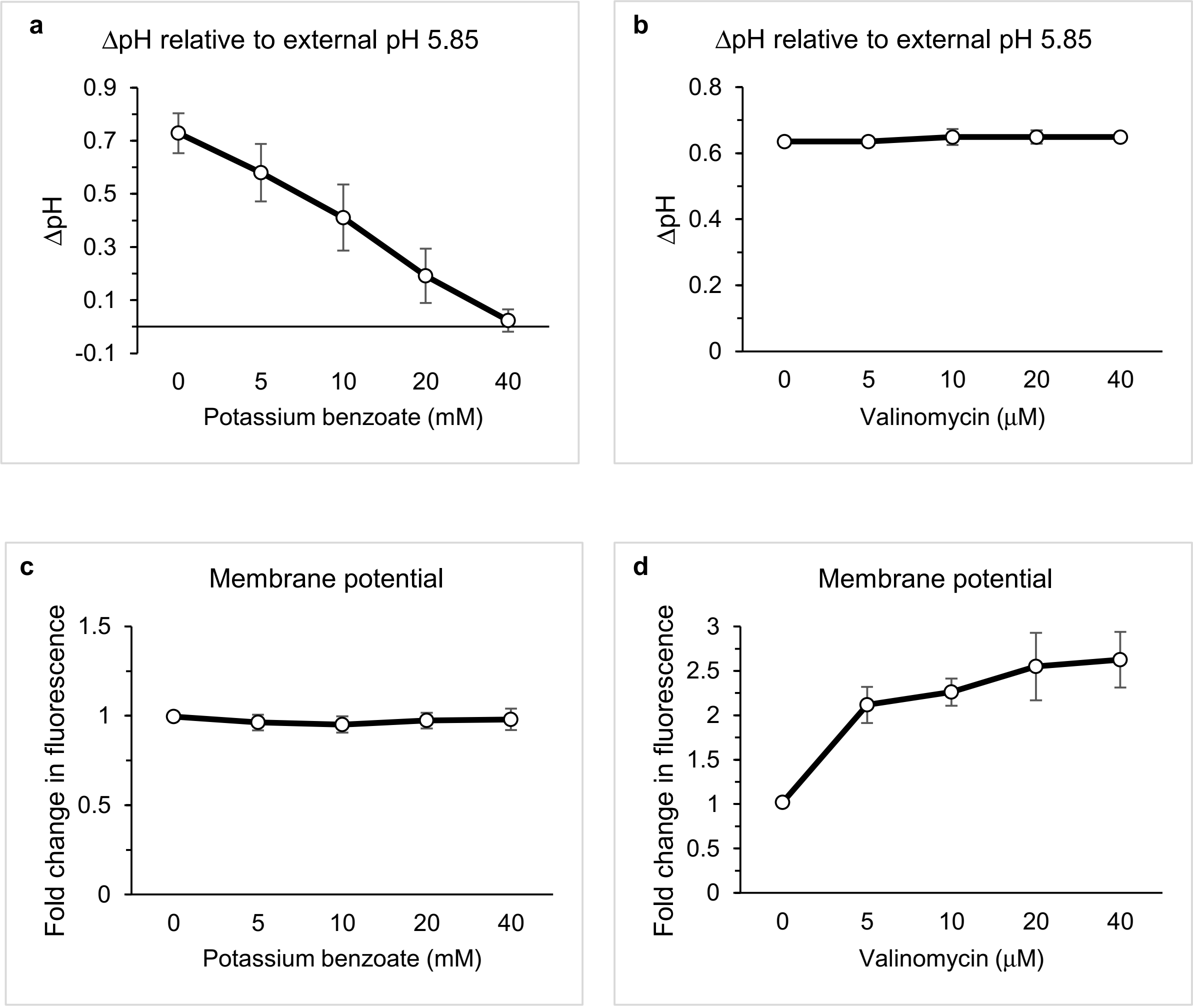

FIG. G. Bacteria incubated in TCSB medium do not respond to benzoate potassium due to the changing external pH. (A) Bacteria were treated with potassium benzoate in TCSB medium. The cell (left) and supernatant (right) fractions were analysed by immunoblotting using antibodies against translocator IpaB. Bacterial numbers were normalized by OD_{600}. Results for WT, Δ*mxiC* and Δ*ipaD* were repeated and gave similar results, other strains were only tested once. (B) External pH was measured when potassium benzoate were added in TCSB containing bacteria at time zero or after 30 min incubation.

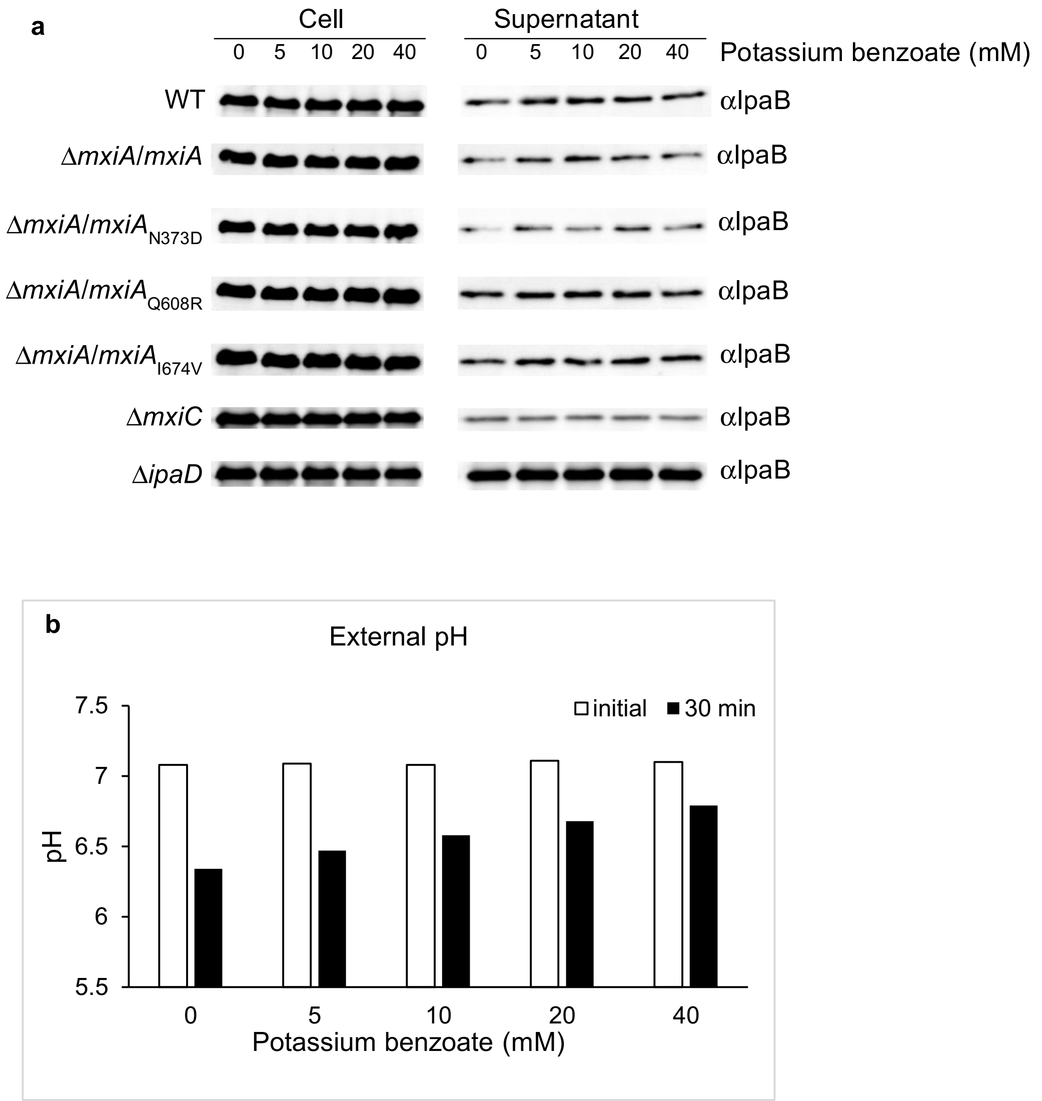

FIG. H. Effect of valinomycin on T3S in *Shigella*. Bacteria were treated with valinomycin in the presence of 150 mM KCl in TCSB medium. The secretion level of IpaB in the absence of valinomycin was set as 100%. Data for WT, Δ*mxiC* and Δ*ipaD* were calculated from two experiments while other strains were only tested once. One representative result is shown in Fig. 3C.

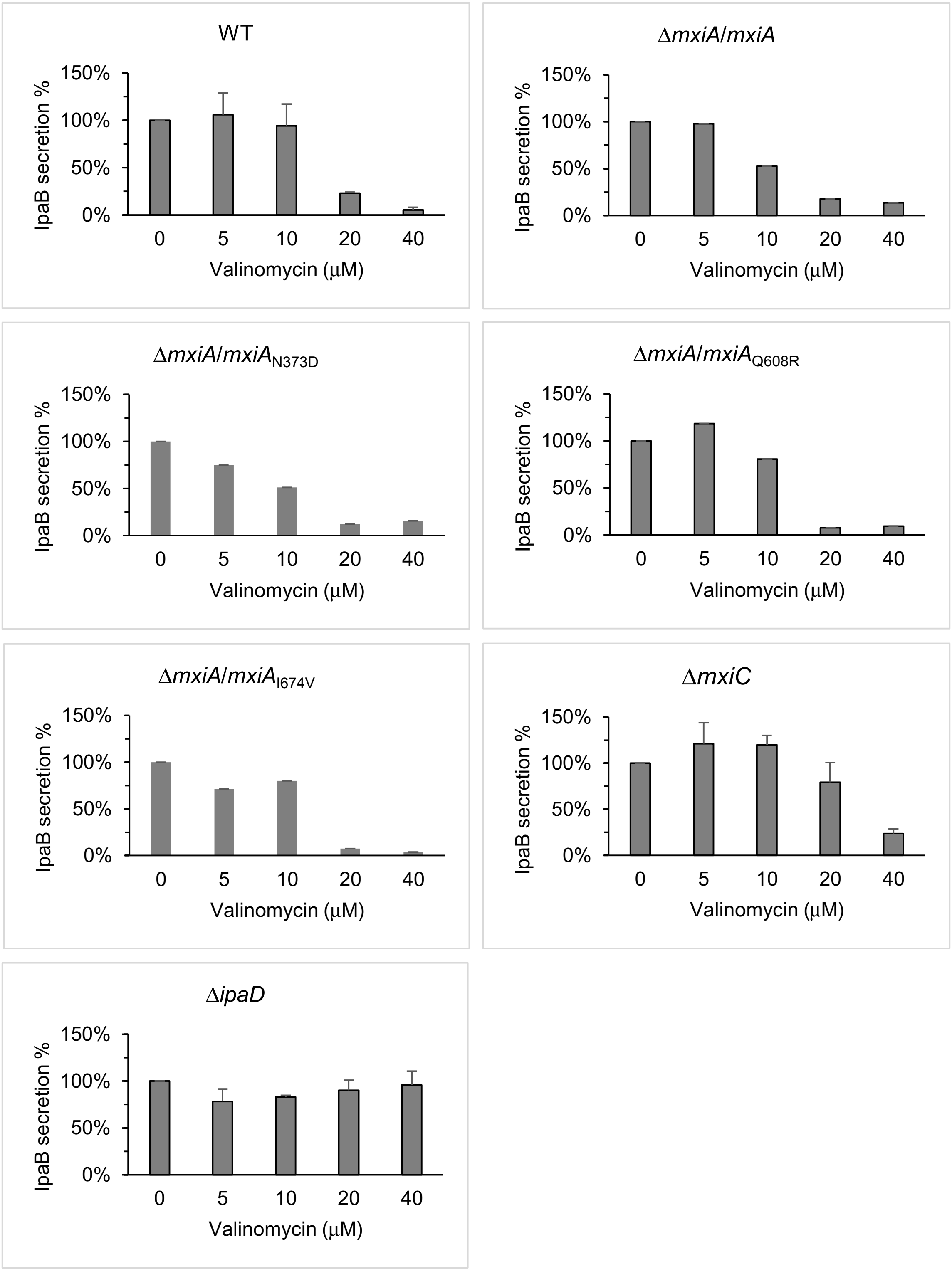

Supplement: S1 File — (PDF) [file pone.0155141.s001.pdf]
